# Protective Effect of Ethyl Pyruvate against Myocardial Ischemia Reperfusion Injury through Regulations of ROS-Related NLRP3 Inflammasome Activation

**DOI:** 10.1155/2019/4264580

**Published:** 2019-01-09

**Authors:** Ji Hae Jun, Jae-Kwang Shim, Ju Eun Oh, Eun-Jung Shin, Eunah Shin, Young-Lan Kwak

**Affiliations:** ^1^Anesthesia and Pain Research Institute, Yonsei University College of Medicine, Seoul, Republic of Korea; ^2^Department of Anesthesiology and Pain Medicine, Yonsei University College of Medicine, Seoul, Republic of Korea; ^3^Yonsei Cardiovascular Research Institute, Yonsei University College of Medicine, Seoul, Republic of Korea; ^4^Department of Pathology, CHA Gangnam Medical Center, CHA University, Seoul, Republic of Korea

## Abstract

Emerging evidence indicates the pronounced role of inflammasome activation linked to reactive oxygen species (ROS) in the sterile inflammatory response triggered by ischemia/reperfusion (I/R) injury. Ethyl pyruvate (EP) is an antioxidant and conveys myocardial protection against I/R injury, while the exact mechanisms remain elusive. We aimed to investigate the effect of EP on myocardial I/R injury through mechanisms related to ROS and inflammasome regulation. The rats were randomly assigned to four groups: (1) sham, (2) I/R-control (IRC), (3) EP-pretreatment + I/R, and (4) I/R + EP-posttreatment. I/R was induced by a 30 min ligation of the left anterior descending artery followed by 4 h of reperfusion. EP (50 mg/kg) was administered intraperitoneally at 1 h before ischemia (pretreatment) or upon reperfusion (posttreatment). Both pre- and post-EP treatment resulted in significant reductions in myocardial infarct size (by 34% and 31%, respectively) and neutrophil infiltration. I/R-induced myocardial expressions of NADPH oxidase-4, carnitine palmitoyltransferase 1A, and thioredoxin-interacting protein (TXNIP) were mitigated by EP. EP treatment was associated with diminished inflammasome activation (NOD-like receptor 3 (NLRP3), apoptosis-associated speck-like protein, and caspase-1) and interleukin-1*β* induced by I/R. I/R-induced phosphorylation of ERK and p38 were also mitigated with EP treatments. In H9c2 cells, hypoxia-induced TXNIP and NLRP3 expressions were inhibited by EP and to a lesser degree by U0126 (MEK inhibitor) and SB203580 (p38 inhibitor) as well. EP's downstream protective mechanisms in myocardial I/R injury would include mitigation of ROS-mediated NLRP3 inflammasome upregulation and its associated pathways, partly via inhibition of hypoxia-induced phosphorylation of ERK and p38.

## 1. Introduction

Timely restoration of coronary flow is essential for myocardial salvage and remains the cornerstone of therapies for myocardial infarction. However, reperfusion inevitably accompanies a specific, sterile inflammation that has been extensively studied to be the principal cause of further myocardial damage and dysfunction [1]. In that context, emerging evidence indicates the role of inflammasome as one of the key regulators of this inflammation-mediated ischemia/reperfusion (I/R) injury [2].

The inflammasome is a multiprotein complex that embodies caspase-1, apoptosis-associated speck-like protein (ASC), and nucleotide-binding oligomerization domain-like receptor with a pyrin domain (NLRP) [3]. As an integral component of the inherent immune system, activation of the inflammasome results in the production of potent inflammatory cytokines, interleukin- (IL-) 1*β* and IL-18, that promote further myocardial damage after I/R [4, 5]. In addition, caspase-1 activation and inflammasome formation lead to a distinct form of cell death called pyroptosis [5]. Accounting for its dominant role in mediating inflammation, experimental endeavors to find therapies targeting the inflammasome in neurodegenerative or metabolic disorders have been made with some promising results [6]. Yet, evidence regarding relevant therapies in myocardial I/R injury is scarce.

Ethyl pyruvate (EP) is a stabilized ester form of the innately existing antioxidant pyruvate [7, 8]. Accordingly, in conjunction with its proven clinical safety, EP conveyed beneficial influences in various inflammatory diseases and I/R injury of major organs [8]. In terms of myocardial I/R injury, EP has been shown to exert potent myocardial protection that is retained even in a hyperglycemic condition [9], which is known to abrogate the protective efficacies of many experimentally proven therapies [10]. However, the underlying mechanisms remain obscure, which include EP's effects of reactive oxygen species (ROS) scavenging and high mobility group box 1 inhibition leading to anti-inflammatory and antiapoptotic effects [11]. Considering that excessive ROS production is a critical activator of the inflammasome [12], EP's protective effects against myocardial I/R injury would theoretically involve regulation of inflammasome activity, which has not been examined heretofore.

This study was aimed at investigating the protective role of EP against myocardial I/R injury and seeking for the mechanistic insights with particular relevance to ROS and inflammasome regulation.

## 2. Materials and Methods

### 2.1. Animal Preparation

All experiments were sanctioned by the committee for the Care and Use of Laboratory Animals, Yonsei University College of Medicine, and were executed conforming to the Guide for the Care and Use of Laboratory Animals published by the US National Institutes of Health (No. 85-23, 1996).

Male Sprague-Dawley rats (10–12 wk old, 250–300 g) were anesthetized with Rompun (10 mg/kg, Vial Korea) and Zoletil 50 (30 mg/kg, Virbac Korea) and were intubated for mechanical ventilation. Normothermia (at around 37°C) was maintained using an electric heating pad. Heparin (200 IU/kg) was given before ischemia intravenously. The right femoral artery was cannulated to monitor mean arterial pressure (MAP). Heart rate (HR) was monitored by subcutaneous stainless steel electrodes.

### 2.2. Experimental Models and Study Groups

Myocardial I/R injury was achieved by encircling the left anterior descending coronary artery (LAD) with a 4-0 silk suture placed through a left parasternal incision [13]. Ischemia was induced for 30 min, confirmed by ST elevation and regional cyanosis of the myocardium, followed by 4 h of reperfusion. In the sham group, the same procedures were performed without LAD ligation. MAP and HR were recorded at baseline, during ischemia, and after reperfusion. EP (50 mg/kg, Sigma-Aldrich, St. Louis, MO) was administered 1 h before ischemia or upon reperfusion via the tail vein, while the I/R-control (IRC) group received an equivalent amount of 0.9% saline (200 *μ*L).

The animals were randomly assigned into four groups: (1) sham (*n* = 10), (2) IRC (*n* = 17), (3) EP-pretreatment + I/R (EP^pre^) (*n* = 17), and (4) I/R + EP-posttreatment (EP^post^) (*n* = 17). Tissues were collected at the end of 4 h of reperfusion.

### 2.3. Myocardial Infarct Size Determination

Upon 4 h of reperfusion, 1 mL of 2% Evans blue dye was administered intravenously while occluding the LAD again. Four to five transverse sections (2 mm thick) of the left ventricle were obtained and incubated with 2% 2,3,5-triphenyltetrazolium chloride (TTC) for 20 min at 37°C, followed by 10 min incubation in 1% TTC. The area at risk was estimated as a percentage of the left ventricle showing the absence of blue staining. The infarct borders were delineated, and the area was quantified with ImageJ software. The infarct size was determined as a percentage of the area at risk.

### 2.4. H&E Staining

After reperfusion, hearts were immediately excised, cross-sectioned, and fixed with 10% buffered formalin. Fixed tissue was then paraffin embedded and sectioned (5 *μ*m) and stained with hematoxylin and eosin (H&E). Five visual fields from each sample block (5 blocks/rat) were randomly chosen and examined by a blind observer using a microscope (×400). A pathologist (E. Shin) who was blinded to all information then evaluated the H&E stained slides for neutrophilic infiltrate, hemorrhage, and acute myocardial necrosis.

### 2.5. Immunohistochemistry

For analyzing the expression of the NLRP3 inflammasome, paraffin-embedded sections from each group were deparaffinized and rehydrated. Then, sections were microwaved in 0.01 M citrate buffer, washed with PBS for 15 min, and subsequently treated with 5% BSA blocking buffer for 30 min at room temperature. Afterwards, these sections were incubated with a primary antibody to anti-NLRP3 (Abcam, UK) for 1 h at 37°C. After washing with PBS for 3 times, a secondary antibody was added, and immunostaining was performed using a DAB kit (Invitrogen, Gibco, USA). The NLRP3-positive area was assessed using ImageJ (US National Institute of Health, Bethesda, MD).

### 2.6. Immunoprecipitaion and Immunoblot Analysis

The left ventricle containing the infarcted area and its neighboring borders was procured. After measuring protein concentrations, 1 mg of protein from each cell lysate was immunoprecipitated using the appropriate primary antibodies and protein G-agarose beads [14]. Proteins were separated on sodium dodecyl sulfate-polyacrylamide gel electrophoresis and immunoblotted with anti-thioredoxin-interacting protein (TXNIP) (Santa Cruz Biotechnology Inc., CA, USA), anti-caspase-1, anti-cleaved caspse-1 (Cell Signaling Technology, Beverly, MA), anti-carnitine palmitoyl transferase 1A (CTP1A), anti-NAPDH oxidase 4 (NOX4), anti-NLRP3, anti-ASC, and anti-IL-1*β* (all Abcam, UK).

### 2.7. Cell Culture

H9c2 cardiomyocytes (ATCC, MD, USA) were maintained in Dulbecco's modified Eagle's medium supplemented with 10% fetal bovine serum, 100 U/mL penicillin, and 100 *μ*g/mL streptomycin (Gibco, USA) at 37°C in 95% humidified air plus 5% CO_2_. Cells were plated into 60 or 100 mm tissue culture dishes with serum-free medium and incubated under normoxic (5% CO_2_ in air) or hypoxic (1% O_2_, 5% CO_2_, and 94% N_2_) conditions. Treatments were as follows: EP (10 mM), MEK inhibitor U0126 (20 *μ*M), or p38 inhibitor SB203580 (10 *μ*M) (Cell Signaling Technology Inc.). We performed hypoxia-induced cellular toxicity using EZ-Cytox-enhanced cell viability assay kit (DoGeN, South Korea).

### 2.8. DNA Constructs and Transient Transfection

The plasmid constructs Myc-tagged TXNIP and GFP-tagged NLRP3 were from OriGene (Rocksille, MD, USA). pcDNA was from Stratagene (La Jolla, CA, USA). Transfection was performed with the *Trans*IT-X2® Dynamic Delivery System (Mirus Bio LLC, USA). In each transfection, 100 ng of expression plasmids were used as indicated. After overnight recovery from transfection, the cells were incubated under a hypoxic condition for 24 h.

### 2.9. Statistical Analysis

All results were expressed as mean ± SD. Hemodynamic data were analyzed by repeated measures of ANOVA. Other variables were analyzed by two-way ANOVA. The *P* values of post hoc tests were adjusted by Bonferroni's methods. The statistical significance of cell examination was analyzed by Student's *t*-test. Values of *P* < 0.05 were considered statistically significant.

## 3. Results

### 3.1. Hemodynamic Parameters

The trends of MAP and HR over time were similar among the groups. MAPs recorded during ischemia were significantly lower compared to their corresponding baseline values in all groups, while HR did not show any significant changes throughout the study period (data not shown).

### 3.2. EP Attenuated Myocardial Infarction and Neutrophilic Infiltration after I/R

**Figure 1:**
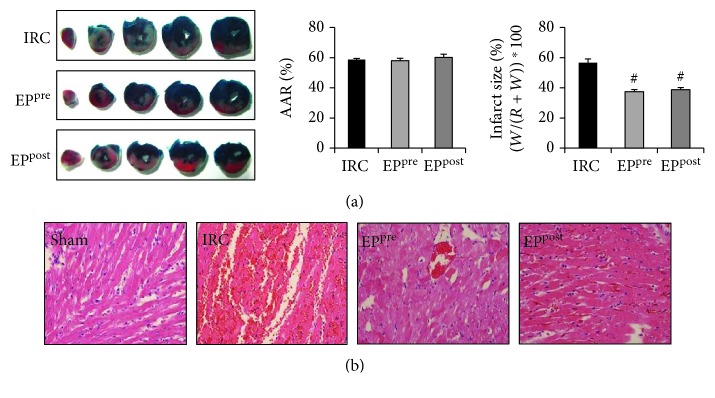
EP attenuated myocardial infarction and apoptosis after I/R. Myocardial infarct size was determined as a percentage of the area at risk using 2,3,5-triphenyltetrazolium chloride (a). Neutrophilic infiltrate, hemorrhage, and necrosis were assessed using hematoxylin and eosin staining (b). EP, ethyl pyruvate; IRC, ischemia (30 min)-reperfusion (4 h) without treatment; EP^pre^, EP (50 mg/kg) treatment 1 h before ischemia; EP^post^, EP (50 mg/kg) treatment upon reperfusion. AAR, area at risk/left ventricle. ^#^*P* < 0.05 compared with the IRC groups (*n* = 5, each).

Myocardial infarct size was significantly decreased in both EP^pre^ and EP^post^ groups compared to those in the IRC group by approximately 34% and 31%, respectively (*P* < 0.05), without any significant intergroup difference between the EP-treatment groups (Figure 1(a)).

EP-treatment groups also displayed reductions in myocardial neutrophilic infiltration, necrosis, and hemorrhage when compared with the IRC group (Figure 1(b)).

### 3.3. EP Ameliorated Myocardial I/R-Induced Increase in NLRP3 Expression

**Figure 2:**
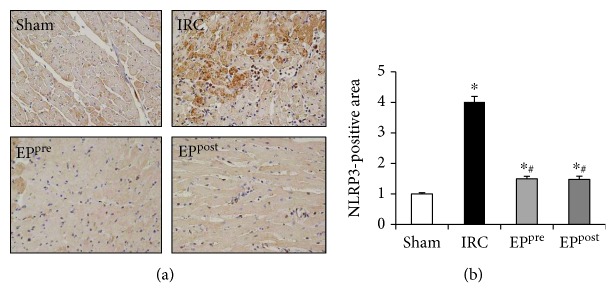
EP ameliorated myocardial I/R-induced increase in NLRP3 expression. Using immunohistochemistry staining, the NLRP3-positive myocardial area following I/R injury was assessed. EP, ethyl pyruvate; NLRP3, nucleotide-binding oligomerization domain-like receptor with a pyrin domain 3; IRC, ischemia (30 min)-reperfusion (4 h) without treatment; EP^pre^, EP (50 mg/kg) treatment 1 h before ischemia; EP^post^, EP (50 mg/kg) treatment upon reperfusion. IRC, ischemia (30 min)-reperfusion (4 h) without treatment in heart. ^∗^*P* < 0.05 compared with the sham group; ^#^*P* < 0.05 compared with the IRC groups (*n* = 5, each).

Using immunohistochemistry staining, the NLRP3-positive myocardial area following I/R injury was assessed. The amount of NLRP3 in the IRC group was significantly increased following I/R injury compared with those in the sham group. Both EP^pre^ and EP^post^ groups exhibited significantly lower NLRP3 expression than the IRC group, without any significant intergroup difference between the EP-treatment groups. However, NLRP3 expression was similar between the EP^pre^ group and the sham group, while it was significantly increased in the EP^post^ group compared with the sham group (Figure 2).

### 3.4. EP Attenuated the Activation of the NLRP3 Inflammasome and Subsequent Increase in IL-1*β* Production following Myocardial I/R Injury

**Figure 3:**
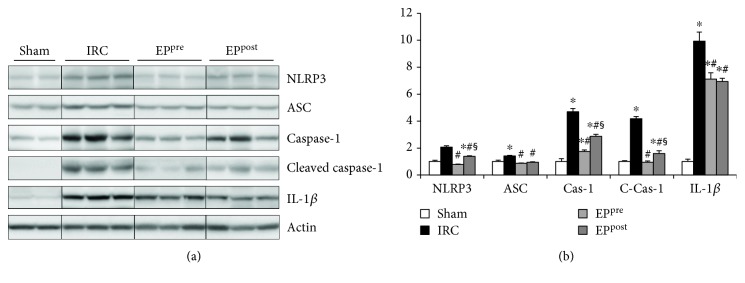
EP attenuated the activation of the NLRP3 inflammasome and subsequent increase in IL-1*β* production following myocardial I/R injury. Immunoblot assays of NLRP3, ASC, caspase-1, cleaved caspase-1, and IL-1*β* expressions in the myocardium after I/R. EP, ethyl pyruvate; NLRP3, nucleotide-binding oligomerization domain-like receptor with a pyrin domain 3; ASC, apoptosis-associated speck-like protein; IRC, ischemia (30 min)-reperfusion (4 h) without treatment; EP^pre^, EP (50 mg/kg) treatment 1 h before ischemia; EP^post^, EP (50 mg/kg) treatment upon reperfusion; IL-1*β*, interleukin-1*β*. ^∗^*P* < 0.05 compared with the sham group; ^#^*P* < 0.05 compared with the IRC groups, ^§^*P* < 0.05 compared between EP^pre^ and EP^post^ (*n* = 7, each).

I/R injury significantly induced the activation of the NLRP3 inflammasome (NLRP3, ASC, caspase-1, and cleaved caspase-1) and the production of IL-1*β* compared to the Sham group. However, these protein expressions could all be significantly attenuated by both EP^pre^ and EP^post^ treatments compared to the IRC group (Figure 3). The EP^pre^ group exhibited significantly lower expressions of NLRP3, caspase-1, and cleaved caspase-1 compared with the EP^post^ group, while the IL-1*β* expression level was similar between the EP-treatment groups.

### 3.5. EP Attenuated Myocardial I/R-Induced Increases of NOX4, CTP1A, and TXNIP

**Figure 4:**
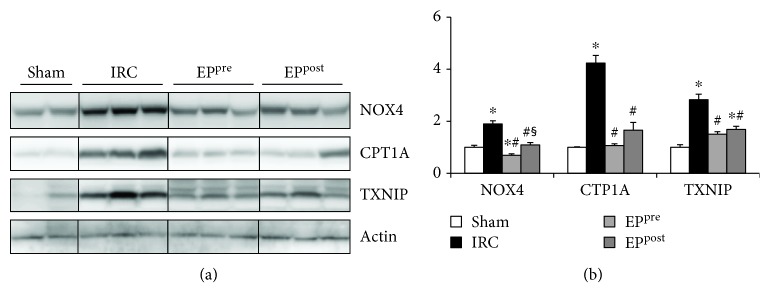
EP attenuated myocardial I/R-induced increases of NOX4, CTP1A, and TXNIP. Immunoblot assays of NOX4, CTP1A, and TXNIP expressions in the myocardium after I/R. EP, ethyl pyruvate; NOX4, NADPH oxidase-4; CTP1A, carnitine palmitoyltransferase 1A; TXNIP, thioredoxin-interacting protein; IRC, ischemia (30 min)-reperfusion (4 h) without treatment; EP^pre^, EP (50 mg/kg) treatment 1 h before ischemia; EP^post^, EP (50 mg/kg) treatment upon reperfusion. ^∗^*P* < 0.05 compared with the sham group; ^#^*P* < 0.05 compared with the IRC groups (*n* = 7, each).

NOX4, CTP1A, and TXNIP are known mediators linked to ROS formation and inflammasome activation. Following myocardial I/R injury, the expression levels of NOX4, CTP1A, and TXNIP were significantly increased in the IRC group compared with those in the sham group, while their elevations were significantly mitigated by both EP^pre^ and EP^post^ treatments compared to those of the IRC group (*P* < 0.05) (Figure 4). NOX4 expression was significantly lower in the EP^pre^ group than in the EP^post^ group, while CTP1A and TXNIP levels were similar between the EP-treatment groups.

### 3.6. EP Decreased Myocardial I/R-Induced ERK and p38 Phosphorylation

**Figure 5:**
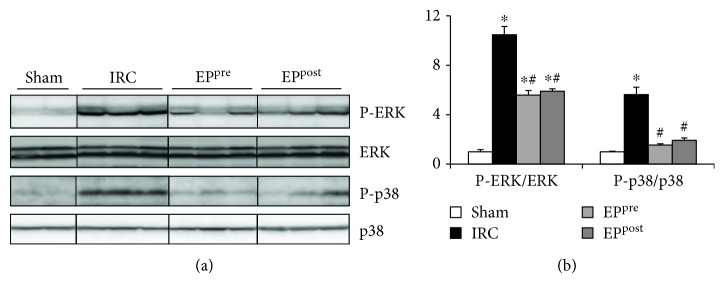
EP decreased myocardial I/R-induced ERK and p38 phosphorylation. Immunoblot assays of P-ERK, ERK, P-p38, and p38 in the myocardium after I/R. EP, ethyl pyruvate; IRC, ischemia (30 min)-reperfusion (4 h) without treatment; EP^pre^, EP (50 mg/kg) treatment 1 h before ischemia; EP^post^, EP (50 mg/kg) treatment upon reperfusion. ^∗^*P* < 0.05 compared with the sham group; ^#^*P* < 0.05 compared with the IRC groups, (*n* = 7, each).

ROS and mitogen-activated protein kinase (MAPK) have been shown to be closely interlinked mutually and with the NLRP3 inflammasome as well [3, 15, 16]. P-ERK and P-p38 levels were increased in the IRC group compared to those in the sham group. Both EP-treatment groups exhibited significantly decreased ERK and p38 phosphorylation compared with the IRC group (*P* < 0.05), without any intergroup (EP^pre^ vs. EP^post^) difference (Figure 5).

### 3.7. EP Reduced Hypoxia-Induced TXNIP-NLRP3 Expressions and their Interaction Partly via ERK and p38 Pathway in H9c2 Cardiomyocytes

To confirm whether the chosen dose of EP (10 mM) lacked cytotoxicity, we examined H9c2 cardiomyocytes cultured for 24 h in the presence or absence of EP under normoxic or hypoxic conditions. 10 mM EP showed no cytotoxicity under both conditions in serum-starved H9c2 cardiomyocytes (data not shown).

**Figure 6:**
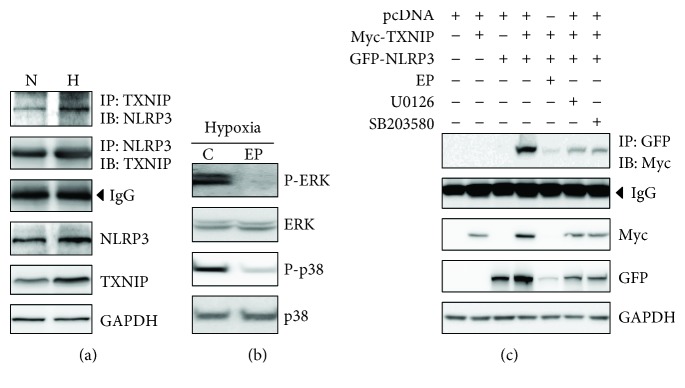
EP reduced hypoxia-induced TXNIP-NLRP3 expressions and their interaction partly via ERK and p38 pathway in H9c2 cardiomyocytes. Immunoprecipitation (IP) and immunoblot (IB) assays of TXNIP and NLRP3 expressions, and their interaction in normoxic (N) or hypoxic (H) conditions in serum-starved H9c2 cardiomyocytes (a). Immunoblot assays of P-ERK, ERK, P-p38, and p38 expressions in a hypoxic condition in serum-starved H9c2 cardiomyocytes treated either with or without EP for 24 h (b). Serum-starved H9c2 cells were transiently transfected with a Myc-TXNIP and GFP-NLRP3 expression plasmids, incubated in the presence or absence of EP for 24 h under hypoxia and subjected to IP and IB analysis (c). EP, ethyl pyruvate; TXNIP, thioredoxin-interacting protein; NLRP3, nucleotide-binding oligomerization domain-like receptor with a pyrin domain 3; U0126, MEK inhibitor; SB203580, p38 inhibitor.

We next examined whether hypoxia induced activations of TXNIP and NLRP3 and their interaction compared to normoxia. Hypoxia clearly increased endogenous TXNIP and NLRP3 protein levels and their interaction compared to normoxia in serum-starved H9c2 cells (Figure 6(a)).

When serum-starved cells were exposed to EP under hypoxia for 24 h, hypoxia-induced increased phosphorylations of ERK and p38 were mitigated (Figure 6(b)).

To discern EP's specific action on the TXNIP-NLRP3 interaction via ERK and p38 under the hypoxic condition, serum-starved cells were transiently transfected with pcDNA, Myc-TXNIP, and/or GFP-NLRP3 expression vectors and then treated with EP or inhibitors as indicated (Figure 6(c)). In TXNIP- and NLRP3-overexpressed cells, the hypoxia-induced increase in the TXNIP-NLRP3 interaction level was significantly attenuated by EP at 24 h after exposure to hypoxia. Likewise, U0126, an MEK inhibitor, and SB203580, a p38 inhibitor, both mitigated the hypoxia-induced increase in the TXNIP-NLRP3 interaction, yet to a lesser degree than that obtained by EP treatment. These results indicate that EP downregulated hypoxia-induced TXNIP-NLRP3 interaction, partly via ERK and p38 pathway.

## 4. Discussion

In the present study, we could corroborate that EP significantly reduced myocardial I/R injury via attenuation of ROS-related regulatory mechanisms linked to NLRP3 inflammasome activation involving NOX4, CTP1A, and TXNIP. EP's efficacy for attenuating myocardial infarction was similar whether it was administered before ischemia or upon reperfusion. In relation to the MAPK signaling pathway, EP's beneficial influence seems to involve mechanisms beyond that of inhibiting hypoxia-induced phosphorylations of ERK and p38.

Although a necessity, restoration of coronary blood flow following interruption and energy deprivation to the myocardium inescapably evokes an inflammatory response that mediates further myocardial damage and dysfunction [17]. Accordingly, extensive research pursued mechanistic insights related to this sterile inflammation after I/R injury identifying key mediators and thus, endowing potential therapeutic targets that may enable mitigating myocardial infarction and enhancing recovery. Among the recognized proinflammatory mediators, recent evidence highlighted the cardinal role of a macromolecular complex consisting of NLRP3, ASC, and caspase-1 called the inflammasome in mediating adverse inflammatory responses after myocardial I/R [18, 19]. Nonetheless, therapeutic measures for myocardial infarction aimed at processes related to attenuating inflammasome activation are limited.

Reperfusion after ischemic insult to the myocardium always accompanies exorbitant ROS formation that induces further myocardial damage [20–22]. Not surprisingly, ROS formation and oxidative stress have been shown to be important promoters of inflammasome activation [23]. In that context, EP seems to be a promising therapeutic agent for myocardial infarction as it exerts a robust antioxidant effect [10, 24, 25], not to mention its clinical safety as it is formed from a naturally occurring metabolic intermediary, pyruvate [26]. Indeed, EP's ability to attenuate myocardial I/R injury has been validated previously [9], while its exact underlying mechanisms remain largely obscure and evidence is lacking regarding its association with inflammasome regulation heretofore. Therefore, we aimed to validate mechanistic insights related to ROS and NLRP3 inflammasome regulation and EP's role in that regard.

As postulated, we could verify that EP's protective efficacy against myocardial I/R involved mitigation of NLRP3 inflammasome activation. Caspase-1 and NLRP3 inflammasome activation results in increased production of IL-1*β*, a powerful proinflammatory cytokine, and in a specific inflammatory cell death referred to as pyroptosis [5]. Indeed, we could verify that myocardial I/R provoked a significant increase in the expressions of NLRP3, ASC, and caspase-1, which comprise the inflammasome complex. We also observed a significantly increased production of IL-1*β* after I/R in the current study. EP administration, whether given before ischemia or upon reperfusion, resulted in significant attenuation of these essential proteins of inflammasome and IL-1*β* compared to the control group. In intergroup comparisons between the EP^pre^ and EP^post^ groups, attenuation of NLRP3, ASC, and caspase-1 expressions by I/R was more significant in the EP^pre^ group. Still, EP's treatment efficacies in terms of its administration in temporal relation to I/R seem to be similar as the degree of IL-1*β* expression, the end product of inflammasome activation, was similar between the EP^pre^ and EP^post^ groups. More definitely, histologic evidence regarding the myocardial infarct size and the degree of neutrophil infiltration showed similar treatment efficacy of EP^pre^ and EP^post^ groups.

To explore detailed mechanistic insights related to ROS formation and inflammasome activation, we further investigated the changes in NOX4, CTP1A, and TXNIP in relation to I/R and EP treatment. NOX4 serves as a source of cellular superoxide anions and has been shown to mediate NLRP3 inflammasome activation via regulation of a key enzyme involved in fatty acid oxidation, CTP1A [27]. In that study, NOX4 deficiency yielded reduced CPT1A expression that consequently resulted in mitigated NLRP3 inflammasome activation in mouse and human macrophages. Thus, the importance of NOX4 and CPT1A as molecular targets for therapies aimed at attenuating inflammasome activation has been implicated in diabetes or metabolic diseases [28–31]. However, there role in myocardial I/R injury, which involves an acute and far more abundant ROS production, in relation to NLRP3 inflammasome activation has never been validated as of yet.

Likewise, TXNIP, a member of the *α*-arrestin protein superfamily, has been shown to bind to thioredoxin thereby inhibiting its ability to scavenge ROS [15, 32]. Thus, being closely related to ROS formation and oxidative stress, TXNIP was identified to be essential for the activation of the NLRP3 inflammasome through direct interaction with NLRP3 via bondage when freed from thioredoxin, which has been verified in myocardial I/R models as well [33].

As our data indicate, expression levels of NOX4 and CTP1A were also significantly increased after I/R as with TXNIP, which could be attenuated by EP treatment regardless of its administration timing. These results confirm the involvement of NOX4 and CTP1A in NLRP3 inflammasome activation for the first time.

Next, we tested the association of MAPK with ROS and inflammasome activation in myocardial I/R. ROS formation was shown to cause the activation of MAPK signaling pathways that consequently results in inflammasome activation in experimental models mimicking inflammatory diseases [34]. In myocardial I/R, ROS-triggered MAPK activation was shown to confer a major role in conveying reperfusion injury [35, 36], while evidence regarding its association with inflammasome activation is lacking. In the current study, activations (phosphorylations) of ERK and p38 were significantly increased by I/R, which could be attenuated by EP treatment.

To validate the association of MAPK activation with TXNIP and NLRP3 inflammasome interaction, we performed additional *in vitro* experiments. Through these, we confirmed that hypoxia clearly increased the endogenous interaction of TXNIP and NLRP3 in serum-starved H9c2 cardiomyocytes. Although we could confirm that EP mitigated the hypoxia-induced phosphorylations of ERK and p38 in the *in vitro* study as well, EP's distinct action mechanism does not seem to be specifically limited to inhibiting the ROS-triggered MAPK activation. This could be verified by our further *in vitro* study showing that EP treatment resulted in far more pronounced inhibition of TXNIP-NLRP3 interaction compared to inhibitors of MAPK pathways (U0126 (MEK inhibitor) and SB203580 (p38 inhibitor)) in hypoxic cells overexpressed with TXNIP and NLRP3.

This study entails the following limitations. First, although we used the same I/R protocols in all experiments, infarct size measurement by TTC staining has its inherent limitation of being affected by the duration of reperfusion [9]. Second, this study did not measure the actual changes in ROS levels according to I/R and EP treatment while EP's antioxidant action in myocardial I/R models, apart from the addressed mechanisms related to inflammasome by the current study, has been well validated through previous studies [24, 25]. Third, concerns exist regarding the difficulty of detecting NLRP3 in cardiomyocytes as opposed to microvascular endothelial cells [2]. Likewise, we could detect NLRP3 in H9c2 cardiomyocytes only after a prolonged period of hypoxia (24 h), which is in agreement with the results of previous studies [37, 38]. In line with this restriction, we could not allow an additional period of reoxygenation as cellular survival was poor in the serum-starved condition, which is necessary for avoiding serum-induced confounders on intracellular signaling. Thus, our *in vitro* results only reflect the hypoxic condition, while our main hypothesis could be clearly validated by our *in vivo* study.

## 5. Conclusions

In conclusion, the current study provides primary evidence that EP's beneficial effect on myocardial infarction involves inhibition of NLRP3 inflammasome activation. We also could firstly confirm that underlying ROS-related mechanisms entail inhibition of hypoxia-induced activations of NOX4, CTP1A, and MAPK pathways, which would hopefully add value to finding new therapeutic molecular targets for myocardial infarction.

## Data Availability

Data will be made available on request.
